# Western white pine SNP discovery and high-throughput genotyping for breeding and conservation applications

**DOI:** 10.1186/s12870-014-0380-6

**Published:** 2014-12-30

**Authors:** Jun-Jun Liu, Richard A Sniezko, Rona N Sturrock, Hao Chen

**Affiliations:** Pacific Forestry Centre, Canadian Forest Service, Natural Resources Canada, 506 West Burnside Road, Victoria, BC V8Z 1M5 Canada; USDA Forest Service, Dorena Genetic Resource Center, 34963 Shoreview Road, Cottage Grove, OR 97424 USA

**Keywords:** Five-needle pine, Genotyping array, Linkage disequilibrium, Marker-based selection, Pedigree reconstruction

## Abstract

**Background:**

Western white pine (WWP, *Pinus monticola* Douglas ex D. Don) is of high interest in forest breeding and conservation because of its high susceptibility to the invasive disease white pine blister rust (WPBR, caused by the fungus *Cronartium ribicola* J. C. Fisch). However, WWP lacks genomic resource development and is evolutionarily far away from plants with available draft genome sequences. Here we report a single nucleotide polymorphism (SNP) study by bulked segregation-based RNA-Seq analysis.

**Results:**

A collection of resistance germplasm was used for construction of cDNA libraries and SNP genotyping. Approximately 36–89 million 2 × 100-bp reads were obtained per library and *de-novo* assembly generated the first shoot-tip reference transcriptome containing a total of 54,661 unique transcripts. Bioinformatic SNP detection identified >100,000 high quality SNPs in three expressed candidate gene groups: Pinus highly conserved genes (HCGs), differential expressed genes (DEGs) in plant defense response, and resistance gene analogs (RGAs). To estimate efficiency of *in-silico* SNP discovery, genotyping assay was developed by using Sequenom iPlex and it unveiled SNP success rates from 40.1% to 61.1%. SNP clustering analyses consistently revealed distinct populations, each composed of multiple full-sib seed families by parentage assignment in the WWP germplasm collection. Linkage disequilibrium (LD) analysis identified six genes in significant association with major gene (*Cr2*) resistance, including three RGAs (two NBS-LRR genes and one receptor-like protein kinase -RLK gene), two HCGs, and one DEG. At least one SNP locus provided an excellent marker for *Cr2* selection across *P. monticola* populations.

**Conclusions:**

The WWP shoot tip transcriptome and those validated SNP markers provide novel genomic resources for genetic, evolutionary and ecological studies. SNP loci of those candidate genes associated with resistant phenotypes can be used as positional and functional variation sites for further characterization of WWP major gene resistance against *C. ribicola*. Our results demonstrate that integration of RNA-seq-based transcriptome analysis and high-throughput genotyping is an effective approach for discovery of a large number of nucleotide variations and for identification of functional gene variants associated with adaptive traits in a non-model species.

**Electronic supplementary material:**

The online version of this article (doi:10.1186/s12870-014-0380-6) contains supplementary material, which is available to authorized users.

## Background

Western white pine (WWP, *Pinus monticola* Douglas ex D. Don) is an economically and ecologically important forest tree species with wide distribution across western North America. WWP faces serious conservation challenges due to its susceptibility to white pine blister rust (WPBR), caused by the exotic fungus *Cronartium ribicola* J.C. Fisch., and its high vulnerability to other disturbance agents including the mountain pine beetle (*Dendroctonus ponderosae*) and fire, both of which are exacerbated by climate change [1]. Today, due largely to WPBR, *P. monticola* exists in fragmented populations that occupy less than 10 percent of this species’ historical landscape [2,3]. Other five-needle pines, such as whitebark pine (*P. albicaulis* Engelm) and limber pine (*P. flexilis* E.James), are subjected to similar conservation challenges [4]. While development of genetic resistance of WWP and other related species to WPBR is underway in several operational programs [5], better understanding of the genetic diversity, population structure, gene flow, and disease and insect resistance of five-needle pines is critical to their proper management, conservation, and restoration.

In the past decade or so, molecular markers have been developed and used to facilitate conservation and WPBR resistance breeding programs [6]. Analysis of amplified fragment length polymorphism (AFLP) markers has revealed that WPBR disease pressure and selection directed by diverging climates have influenced genetic diversity among WWP populations in different geographical regions [7-9]. Several AFLP markers have been shown to be tightly linked with WWP major gene (*Cr2*) resistance against WPBR [10]. More recently, nucleotide diversity has been investigated through PCR-sequencing of candidate genes under adaptation of host defense response [11]. Progress in association genetics has led to the identification of single nucleotide polymorphism (SNP) and simple sequence repeat (SSR) markers of a few candidate genes associated with quantitative disease resistance traits [12,13]. Despite these advances, the application of genomic resources, such as high-throughput markers (SNPs and SSRs) and genotyping arrays, remains scarce for WWP and other five-needle pines as these species are quite evolutionarily distant from the few conifers with available draft genome sequences and related genomic information [14,15].

To develop effective, long term management strategies for WWP and WPBR, ongoing research is needed to improve understanding of the influence that climate and environmental factors have in changing and shaping *P. monticola* populations. To achieve this objective and realistically score individual genotypes using inexpensive high-throughput techniques, a large number of molecular markers that are easy to score on a large number of WWP populations are needed. While SNP markers are abundant in the genome and have the potential to be excellent tools for these research objectives, to date there is no SNP database or SNP arrays available for WWP.

Next generation sequencing (NGS) strategies for high-throughput SNP discovery and genotyping include restriction-site-associated DNA tags - RAD [16], genotyping by-sequencing - GBS [17], and multiplexed-shotgun genotyping - MSG [18]. RNA-seq is also an important genomic technology for discovery of a large number of DNA markers, including SNP and SSR at transcriptome level. Because RNA-seq produces short cDNA sequence reads targeting at exoms and mainly at protein coding regions, DNA variations associated with phenotypic traits are more easily linked to biological roles for functional characterization of candidate genes than would occur using genomic DNA-based approaches. RNA-seq has wide application to ecological and evolutionary research and it is well suited to understanding speciation and eco-type-specific adaptation by revealing differences in gene expression patterns between populations [19]

The objective of this study was 1) to characterize the transcriptome of tree shoot tissues from resistance germplasm, 2) to develop SNP markers based on a candidate gene approach, and 3) to apply high-throughput SNP genotyping to the reconstruction of pedigrees and resistance screening in WWP conservation and breeding programs. We used RNA-seq for SNP discovery in the transcriptome *de-novo* assembled from shoot-tip tissues based on bulked segregation of major gene resistance (*Cr2/-*) and susceptibility (*cr2/cr2*) to *C. ribicola*. The SNP assay was designed based on candidate genes related to disease resistance and Pinus highly conserved genes (HCGs). Those SNPs validated here by high-throughput genotyping in a collection of resistance germplasm improve the genomic tools available for WWP and other five-needle pines.

## Results

### *De-novo* assembly of shoot-tip transcriptome

Construction of six cDNA libraries from pooled RNA samples representing WPBR resistant and susceptible genotypes enabled us to generate and gain a global view of the transcriptome in the shoot tip tissues of *P. monticola*. A total of 348.6 million 100-bp paired-end reads were collected from the six cDNA libraries, which represents sequencing data of approximately 33.2 to 89.8 million paired-end (PE) reads per library. A total of 95,727 unique contigs with N50 of 920-bp and average length of 630-bp were produced by *de-novo* assembly with 123 million RNA-seq 100-bp PE reads from three cDNA libraries constructed from resistant tissues (Additional file 1: Table S1).

54,661 transcripts were extracted from the assembly with read count ≥ 50 per contig, or read count < 50 per contig but with BLASTn E < 10 e-10 when searched against the Pinus Gene Index (PGI) database (Additional file 1: Table S1). All these contigs were used as the shoot-tip reference transcriptome for further analysis, which had a total length of 46 Mb, N50 of 1,376-bp, and average length of 843-bp (Additional file 1: Table S1). BLASTn analysis of the shoot-tip reference transcriptome revealed that it contained 21,930 contigs (40.10% of the total) as Pinus HCGs, since they showed identical hits (E values < 10 e-100) to the PGI database. From this reference transcriptome, a total of 41,460 proteins were predicted by TransDecoder with minimum protein length of 50. Of all putative proteins, 14,287 (30.7% of the total) were putatively complete protein sequences (Additional file 1: Table S2). The WWP shoot-tip reference transcriptome with 54,661 contigs has been deposited at DDBJ/EMBL/GenBank under accession GBQX01000000.

Of 41,460 putative proteins derived from WWP shoot-tip reference transcriptome, 79.4% and 61.5% of them showed significant similarity to the PGI database and loblolly pine (*P. taeda*) genome database respectively (tBLASTn or BLASTp with E < 10e-6). tBLASTn search of *P. taeda* protein database (including 64,809 putative protein sequences) against WWP sequences revealed that 92.9% of them had significant homology hits (E < 10e-6) in WWP shoot-tip reference transcriptome (Additional file 1: Table S3). In contrast, only 830 WWP shoot-tip transcripts (1.5% of total) showed identical hits in the poplar leaf rust fungus (*Melampsora laricis-populina*) genome (BLASTx with E value < 10 e-100), suggesting rare fungal infections in the resistant tissues (Additional file 1: Table S3).

### Gene annotation

Gene Ontology analysis was performed for 54,661 transcripts in the WWP shoot-tip reference transcriptome using BLAST2GO, 56.7% of them showed significant BLASTx hits in the NCBI nr database. All BLAST top-hit species were plants except one fungal species *Botrytis cinerea*, and *Picea sitchensis* accounted for 24.9% of the total contigs while *B. cinerea* accounted for only 0.2% of the total contigs (Additional file 2: Figure S1), suggesting that contamination was not a serious problem in the data set of WWP shoot-tip reference transcriptome. 26,831 contigs (49.1% of the total) were assigned to at least one GO term, and 6,327 of them encoded for putative enzymes. As compared with WWP primary needle reference transcriptome [15], significant enrichment of a series of GO term categories was found and in general, sequences under these categories were significantly over-represented in the shoot-tip tissues (Additional file 3: Figure S2). For example, under category of “response to biotic stimulus”, 1170 genes were expressed in shoot-tip tissues and only 465 genes were expressed in primary needle tissues, suggesting difference of basal defense between these two types of WWP tissues.

Seven hundred and forty-five contigs in the shoot-tip reference transcriptome were identified as resistance gene analogs (RGAs) encoding proteins with domains of nucleotide-binding site and leucine-rich repeats (NBS-LRR) by BLASTx search against 128 WWP RGAs cloned previously [10]. A set of differentially expressed genes (DEGs) was identified in *P. monticola* needle tissues in host defence in response to *C. ribicola* infection at early stage [15], 740 of them were detected to be expressed in shoot-tips. We selected genes of these three groups HCGs, RGAs, and DEGs as reference sequences in mapping of RNA-seq reads for further SNP discovery.

### SNP discovery and characterization

**Figure 1:**
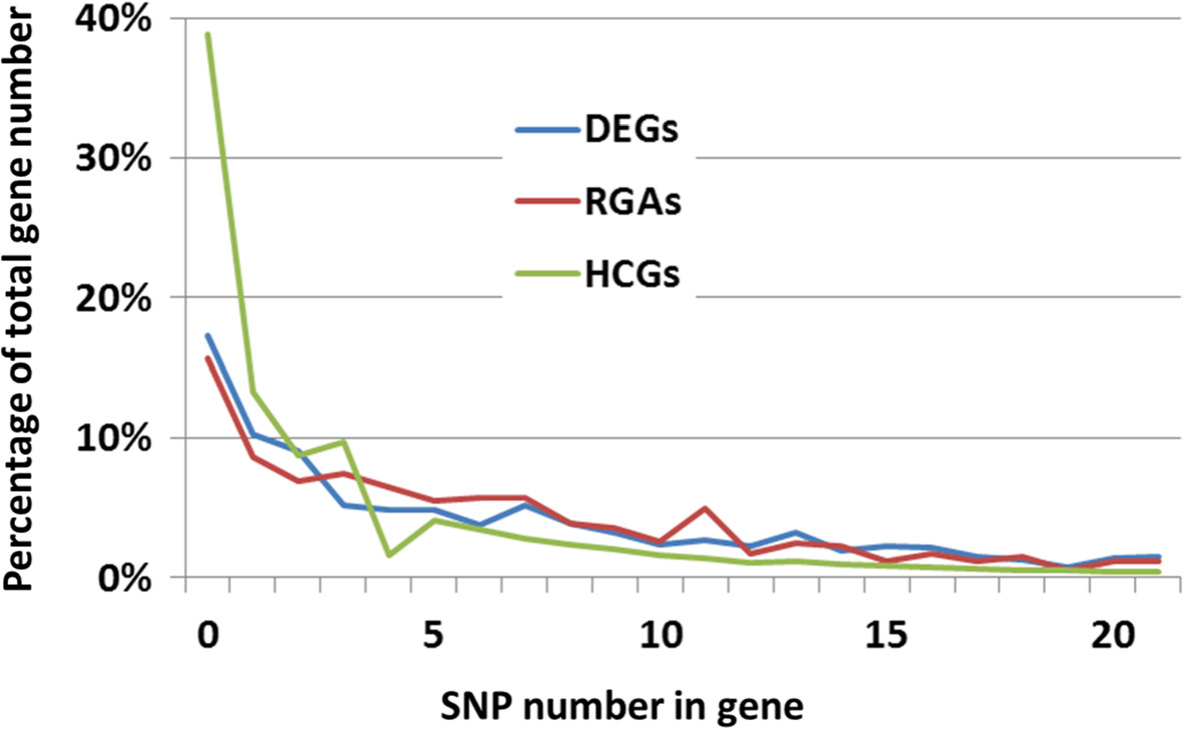
**Distribution of single nucleotide polymorphism (SNP) in contigs of three groups of candidate genes expressed in the shoot-tip tissues.** HCGs: Pinus highly conserved genes, DEGs: differential expressed genes in plant defense response, RGAs: resistance gene analogs.

Using CLC Genomics Workbench 5.1 to map PE reads of the six cDNA libraries to the reference sequences, 2,043 indels, 2,857 multi-nucleotide variants (MNV), and 104,452 bi-allelic SNPs were mapped to 41,460 putative protein-coding regions, 57,139 SNPs (54.7%) resulted in an amino acid change (nonsynonymous SNP). We also detected 97,063 SNPs in the HCG group, 7,248 in DEG group, and 6,078 in the RGA group (Additional file 1: Table S4). These SNPs, which totalled 106,399, were distributed across 14,730 contigs with one SNP per 263-bp on average in three candidate groups. HCGs showed the lowest SNP density at one SNP per 285-bp (0.35%). DEGs and RGAs had intermediate and high SNP densities at one SNP per 126-bp and 81-bp (0.79% and 1.23%) respectively. Polymorphic genes accounted for 61%, 83%, and 84% of the total genes in the candidate groups of HCGs, DEGs, and RGAs respectively (Figure 1, and Additional file 1: Table S4).

**Figure 2:**
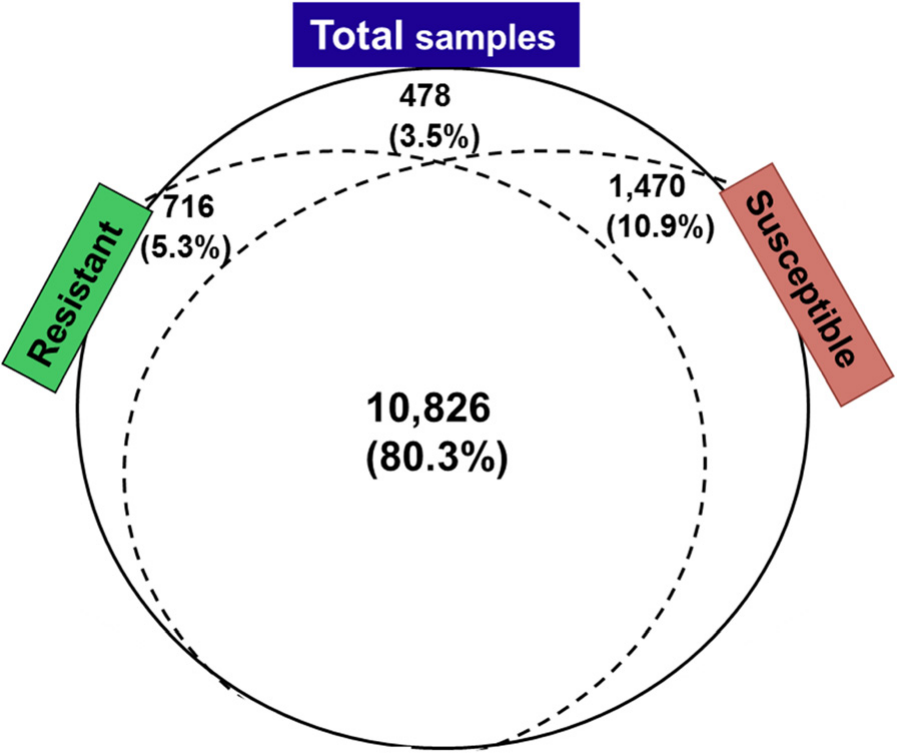
**Unique and shared single nucleotide polymorphism (SNP) of** ***Pinus*** **highly conserved genes (HCGs) within resistant and susceptible seedlings.**

A total of 13,490 HCGs were polymorphic. A detailed examination of SNP distribution revealed that 80.3% (10,826) of these HCGs were polymorphic in both resistant and susceptible samples while 10.9% (1,470) of HCGs were found to be polymorphic only in susceptible seedlings and 5.3% (716) of HCGs were found to be polymorphic only in resistant seedlings. The remaining 3.5% (478) of polymorphic HCGs were homozygous but their alleles were different between resistant and susceptible samples (Figure 2). SNP sites present only in resistant or only in susceptible seedlings were considered the highest priority SNP sites for genotyping verification to identify resistant trait-associated DNA markers.

### SNP genotyping

Two different genotyping assays tested a total of 432 *in-silico* SNP loci in 376 seedlings by the Sequenom iPlex technology. Within the first (1st) SNP array, nine genomic DNA samples were removed from genotyping analysis due to too many missing data, resulting in a sample size of n = 179. Analysis of each SNP locus for the three genotypes (A/B/H) found that 301 SNPs (69.7%) were successfully genotyped while the remaining 131 SNPs (30.3%) were scored as ‘failed’ due to missing genotype data in more than 20% of all samples; poor PCR amplification and low signal intensities resulted in missing data.

As summarized in Table 1, out of the 301 SNP loci that were genotyped with a signal, 74 (24.6%) were monomorphic and the other 227 (75.4% of the 301 genotyped SNPs) were verified as polymorphic among the genotyped samples (sequences of their primers and probes are listed in Additional file 4: Table S5). For each SNP locus, observed (Ho) and expected levels of heterozygosity (He) under Hardy–Weinberg equilibrium (HWE), and significance level for the test for departures from HWE, are shown in Additional file 4: Table S6. A large proportion of SNP markers, 45 in the 1st array and 33 in the second (2nd) array, were identified to be deviated significantly from HWE at *P* < 0.05 with Bonferroni-correction, probably due to breeding selection of the resistant germplasm from natural populations.

**Table 1:** **Characteristics of** ***in-silico*** **SNPs subjected to verification by high-throughput genotyping**

**Gene groups**	**Total**	**Good SNP loci**		**Polymorphic SNP loci**		
		**no. (%)**	**MAF = 0 (n)**	**0 < MAF < 0.05 (n)**	MAF ≥ 0.05 (n)*	**SNP marker in total (%)**
**HCGs**	162	118 (72.8%)	19	3	96	61.1%
**DEGs**	118	87 (73.7%)	20	3	64 (6)	56.8%
**RGAs**	152	96 (63.2%)	35	6	55 (6)	40.1%
**Total**	432	301 (69.7%)	74	12	215 (12)	52.50%

Note (*): 12 SNP loci (six in RGAs and six in DEGs) showed 100% heterozygocity with MAF values at 0.5.

The distributions of minor allele frequency (MAF) and Ho for the polymorphic loci were similar in two genotyping arrays (Additional file 5: Figure S3 and Additional file 6: Figure S3). The mean Hos for all 227 polymorphic SNP markers were estimated to be 0.529 ± 0.2414, and 0.446 ± 0.178 for the 1st and 2nd SNP arrays, respectively. The candidate group of HCGs had the highest successful rate for conversion of *in-silico* SNP loci into SNP markers (61.1%) while this rate was only 40.1% for the candidate group of RGAs. In total, 215 SNPs showed a MAF > 0.05 in the sets of tested seedlings. The twelve SNPs that had the highest Ho level of 100% were excluded for population genetics analysis. Thus, a final genotypic data set consisting of 203 SNP loci was used for pedigree reconstruction and LD analysis.

### Population structure and full sibship reconstruction

**Figure 3:**
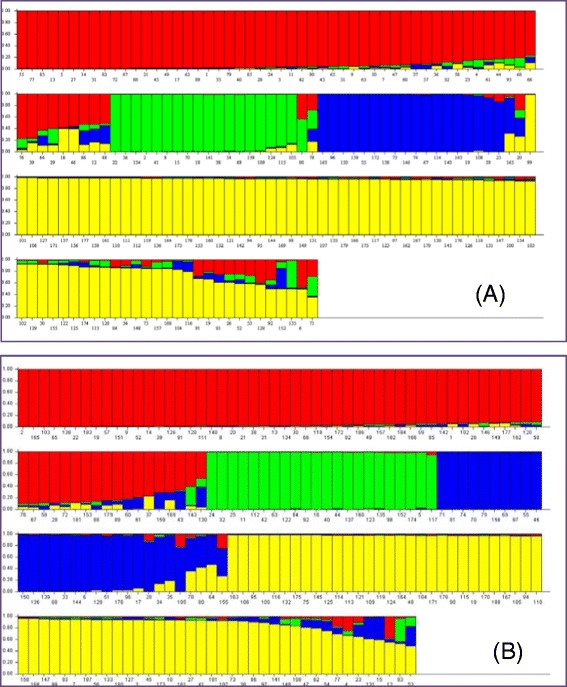
**Genetic diversity among** ***Pinus monticola*** **germplasm by bar plot representation of the percentage of the gene pool in each genotyped seedling. (A)** 179 seedlings genotyped by 108 SNP markers in the 1st SNP array; and **(B)** 188 seedlings genotyped by 95 SNP markers in the 2nd SNP array.

Principal component analysis (PCA) showed that the first three principal components explained approximately 60% of the total variation and clear ancestry clusters displayed within the collected samples (Additional file 7: Figure S4). Investigation of population structure with the model-based Bayesian clustering method in STRUCTURE showed that the most likely number of clusters (K) was 4 using the ΔK calculation (Additional file 8: Figure S5). Four genetic clusters were consistently uncovered by two different sampling in the resistant germplasm in the 1st and 2nd SNP arrays (Figure 3).

Using COLONY to reconstruct sibship and parentage by the most accurate method of full-likelihood, we found that 179 seedlings in the 1st SNP array and 188 seedlings in the 2nd SNP array were assigned into 35 and 36 full-sib seed families respectively. Both SNP assays revealed the three most abundant seed families, each of which accounted for >10% of the total genotyped samples (Additional file 2: Table S7). These results were largely supported by the known pedigrees and origins of these seedlings in the resistance germplasm collected from breeding programs. The seed family with least members was assigned with only one seedling.

### Linkage disequilibrium (LD) analysis

**Figure 4:**
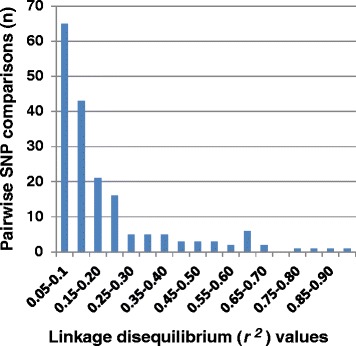
**Distribution of the level of significant linkage disequilibrium (** ***r*** ^***2***^ **) calculated by pairwise comparison of single-nucleotide polymorphisms (SNPs).** Only 1.6% of the total SNP comparison pairs are shown here with statistical significance using a Bonferroni correction for multiple tests.

A total of 11,139 SNP pairs were compared for LD estimates. Chi-squared tests (at *P* < 0.05) showed significant LD estimates for 962 SNP pairs (8.6% of total), but this pair number was reduced to 183 (1.6% of the total) with an average LD estimate at *r*^*2*^ = 0.2 after a highly conservative Bonferroni correction for multiple tests (Figure 4). When major gene resistance genotypes (*Cr2/-* vs. *cr2/cr2*) were considered in the LD analysis, we detected 21 SNPs (each from one unique gene) in significant LDs with *Cr2*. After Bonferroni correction, six genes still showed significant LDs with *Cr2*, including three RGAs (two NBS-LRR genes and one RLK gene), two HCGs, and one DEG (Table 2). Despite not knowing their genetic distances, SNP loci with significant LD may share locations on the same chromosomes. The SNP of the DEG A05_contig_4105 was shown to be tightly associated with major gene (*Cr2*) resistance (*r*^*2*^ = 0.81, *P* = 2.6 E-39). For this SNP marker, CC, GC, and GG genotypes accounted for 26.1%, 68.5%, and 5.4% of the total resistant seedlings; and 0%, 4.6%, and 95.4% of the total susceptible seedlings. This SNP locus thus is an excellent marker for *Cr2*-resistance selection across four populations in WWP germplasm.

**Table 2:** **Identification of SNP loci in significant linkage disequilibrium (LD) with major gene (** ***Cr2*** **) resistance**

**Contig-SNP IDs**	**R^2**	**pDiseq**	**N**	**Candidate group**	**SNP array**
A05_contig_4105	0.808898	2.62E-39***	179	DEG	1st
F0_contig_48562	0.080057	2.29E-04*	179	RGA	1st
F0_contig_3186	0.140014	3.71E-07***	188	RGA	2nd
F0_contig_9161	0.072281	2.87E-04*	188	RGA	2nd
F0_contig_29965	0.067681	4.10E-04*	188	HCG	2nd
F0_contig_3704	0.067235	4.21E-04*	188	HCG	2nd

p values *P < 0.05, ***P < 0.001 after Bonferroni correction.

## Discussion

### SNP discovery by a modified RNA-seq approach

Without requirement of pre-existing genomic sequence data, RNA-seq has been shown to have an increasing range of applications in the discovery of novel genes, transcripts, RNAs, alternative splice junctions, fused sequences, and nucleotide variations (such as SNP and SSR) in non-model species [20-23]. By integrating regular RNA-seq with bulked segregation analysis, we demonstrate that this approach is an effective strategy for selecting SNPs with high potential to identify DNA variations associated with adaptive traits at transcriptome level in WWP. A recent study found that 15 individuals were needed for accurate allele frequency prediction by RNA-seq approach [24]. Coincidentally, our work used six bulked samples (each pooled from 15 individuals) and recovered a total of ~100,000 high quality SNPs by mapping of 348.6 million RNA-seq reads against three sets of candidate genes under a series of stringent detection criteria. Availability of these novel *in-silico* SNPs would help provide a large amount of DNA markers for breeding and conservation programs of this important conifer species.

The Sequenom iPlex has been reported as one of highly reliable high-throughput SNP genotyping platforms with wide applications [25,26]. We adapted it for WWP SNP genotyping due to a more cost-effective and flexible nature of this technology. SNP marker conversion rates from *in-silico* SNPs to validated loci have been reported for maritime pine (*P. pinaster*) (42.5%), lodgepole pine (*P. contorta var. latifolia*) (30.0%), Aleppo pine (*P. halepensis* Mill.) (76.6%), and Douglas fir (72.5%) [27-30]. The present study revealed an average conversion rate of 52.5% in *P. monticola*. The HCG group showed a much higher conversion rate of 61.1%; this rate is comparable to those SNPs mined by genomic re-sequencing in other tree species [31,32]. Variation in SNP marker conversion rates suggests criteria for *in-silico* SNP selection and genotyping design, as well as types of genotyping platforms are important. For example, the *in-silico* SNP-mining process with stringent quality criteria can distinguish sequence variations from sequencing artefacts. It is possible that the rate of conversion of *in-silico* SNPs can be improved even more in WWP by optimizing primer design and PCR amplification conditions because we found that some iPlex failed SNPs could be genotyped properly by qPCR genotyping methods such TaqMan and HRM (Liu, unpublished data). Identification of exon-intron boundaries by exome sequencing will improve design of SNP genotyping arrays. Furthermore, as compared to sample-pooling strategy, SNP detection by NGS on individual samples, especially on haploid megagametophyte samples in conifer, has potential to increase overall confidence for *in-silico* SNP detection.

Our work demonstrates how combining bulked segregation-based RNA-seq with high-throughput SNP arrays enables fast, cost-effective, and yet reliable identification of the most informative (population-specific) markers among hundreds of thousands of *in-silico* SNPs. We believe that this cost-effective approach for detecting the most informative SNPs can be readily adapted and applied to other non-model conifers, including five-needle pine species (e.g., *P. albicaulis* Engelm and *P. flexilis* E.James),

### Candidate gene-based SNP array

In the present study we demonstrated the utility of candidate-based approach for selection of a subset of available *in-silico* SNPs: first, RNA-seq-based transcriptome profiling identified WWP candidate genes (e.g., RGAs and DEGs) having potential biological functions in genetic resistance and host defense against attack by pests, pathogens, and environmental stresses; second, transcriptome profiling also revealed highly conserved genes, even orthologous genes, in conifer species [15]. Because RGAs and DEGs are excellent targets for investigating plant-microbe-environment interactions and HCGs are the most favourable choices for comparative genomics study across related taxa, then we selected SNPs of these candidate groups to develop high-throughput genotyping assays. While SNPs represent a genetic variability of individual at the finest level, if a significant number of SNPs are available, it is not necessary to genotype all the available SNPs throughout the whole genome. Selection of a subset of SNPs that is sufficiently informative but still small enough for the best balance of affordable cost and research objectives is an important step toward effective association studies and genomic selection [33].

A few candidate gene-based case studies have found SNPs and haplotypes associated with quantitative traits in conifers [11,34] and in other plant species [35-40]. Using LD analysis, in this study we identified a defense-responsive gene A05_contig_4105 as being one associated with the *Cr2* gene (Table 2). A05_contig_4105 encodes an F-box protein that has high homology with the *P. taeda* protein AEW08082 and its expressed transcript was specifically up-regulated in the primary needles of resistant seedlings after *C. ribicola* infection [15]. F-box proteins contain at least one F-box domain that is commonly linked with other motifs such as LRRs and tryptophan-aspartic acid (WD) repeats for protein–protein interactions associated with signal transduction networks and other cellular functions [41].

Despite the disadvantages of relatively low read-mapping coverage and high polymorphism levels, we included *in-silico* SNPs of 152 RGAs in the Sequenom iPlex assay. Genotyping of RGA SNPs is more likely to identify genetic associations with disease resistance traits due to their putative functions in plant innate immune systems. Plant NBS-LRR and RLK proteins mainly function in host resistance by specific interactions with pathogen effectors, which trigger plant defense responses that inhibit pathogen growth and spread inside infected tissues [42]. We previously identified over one hundred RGAs of the NBS-LRR and RLKs in *P. monticola* by genomic PCR cloning and several RGA-related AFLP markers linked to *Cr2* in genetic mapping populations [10]. Here we revealed that 175 unique RGA transcripts were expressed in the shoot-tip tissues and ~ 2,000 *in-silico* SNPs were identified in their sequences. Of 96 RGAs genotyped successfully, 61 of them showed polymorphism (Table 1). Three polymorphic RGAs were identified in significant association with major gene (*Cr2*) resistance by LD analysis in the genotyped populations (Table 2). The RGA F0_contig_3186 encoded a putative RLK protein with highest homology to the *Picea glauca* protein ABF73316.1 (expect E = 0.0), and another two RGAs, F0_contig_48562 and F0_contig_9161, encoded NBS-LRR proteins. Additional SNPs, especially those non-synonymous SNPs in the above mentioned three RGAs, would provide both positional and functional variation sites for further characterization of major gene resistance against *C. ribicola*. The large amount of SNP markers, especially those SNPs in the candidate genes, may prove useful to study the evolution and adaptation of resistance mechanisms under selection pressure of climate change and WPBR in the native white pine populations across North America. In the future we will conduct sequence comparison and subsequent functional characterization of resistant and susceptible haplotypes of the related NBS-LRR and RLK genes to determine if any of these RGAs is responsible for the *C. ribicola*-resistance phenotype.

### Identification of SNP markers by LD analysis for resistance screening

Discovery of a large number of SNPs along genome using NGS followed by genotyping of a set of samples with available phenotypes has become standard practice for fine genetic mapping of complex traits. In this study, we used a collection of WWP resistant germplasm to investigate genotype-phenotype relationships. LD, which is the non-random co-segregation of alleles at two loci, can result from many factors, including effective population size and structure, recombination rate, genetic drift, mating system, and selection [43]. Recombination between homologous chromosomes causes LD to decay as the distance between two loci increases during meiosis. In general, LD decay is faster in open-pollinated plants and in more diverse populations of the same species, but rates of LD decay may vary greatly in different genes and genomic regions in the same species [44]. Thus, information on LD content is a crucial prerequisite for any genome-wide association study to fine-tune both targeted genomic regions and candidate genes.

As monoecious gymnosperms, Pinus species show LD decay rates of ~500 to 2,000 bp [45]. Due to this pattern of rapid LD decay in conifers, genetic associations revealed by SNPs are likely to be located in close proximity to causative polymorphisms [34]. Our previous studies showed an intragenic LD decay to *r*^*2*^ estimate of 0.3 within 600 ~ 700-bp in *P. monticola* DEGs [11-13], suggesting that related candidate genes may have a high resolution for association studies. In the present study, at least one SNP marker was found to be tightly associated to *Cr2* with high LD in the tested germplasm across four populations with as many as 35 full-sib families (Table 2, Figure 3, and Additional file 2: Table S7). We suggest that these nucleotide variations may be used as selectable markers for breeding WWP with major gene resistance to *C. ribicola*. Other SNP markers of the RGAs and DEGs with significant LDs (Table 2) may also be very close to, or within, the gene affecting the resistance trait. To confirm this hypothesis we will conduct a continuous study to determine the extent of inter- and intra-chromosomal LD using WWP genetic mapping populations. Association mapping using a genome-wide approach still requires accumulation of sufficient genomic resources in five needle pines.

### Population structure of WWP resistant germplasm

Lack of genetic diversity and ecological challenges (e.g., habitat destruction and environmental change) are two causes of population reduction and species extinction. Conifer seed orchards are commonly used to produce consistent, abundant, and genetically improved seeds with well-adapted environmental performance. These orchard seed lots are used for reforestation and restoration activities with species like WWP. Unfortunately, orchard seed lots are usually composed of undetermined proportions of seeds contributed by many parents through out-crossing and open pollination. Furthermore, it is critical that appropriate levels of genetic diversity are maintained to avoid inbreeding and loss of rare alleles by genetic drift in forest seed orchards or seed collections. While elite seed orchards can be developed by pyramiding favorable alleles, favourable alleles may be dispersed in different stands/ancestors. Complete pedigree information is thus an essential prerequisite for the selection and deployment of elite genotypes in modern conservation and breeding [46]. Molecular-based parentage analysis has been used to quantify genetic diversity and to help prevent inbreeding in reforestation stocks [47,48]. Maintenance of genetic diversity in reforestation stock of long-lived tree species such as WWP is key to helping ensure the continued presence of this species in forests and forested ecosystems.

Sibship reconstruction in our study provided the clearest evidence for seed family structure in a collection of WWP germplasm. Accuracy of parentage analysis increases with the number and diversity of genetic loci. Popular parentage inference methods (e.g., Colony) can be applied with confidence in natural populations with highly polymorphic loci [49]. SNPs are powerful for parentage inference and a previous study suggested that 60–100 SNPs may allow accurate pedigree reconstruction in large managed and/or natural populations [50]. We took careful consideration of the number and quality of SNP markers to increase the accuracy of our parentage assignments. WWP parentage assignment and pedigree reconstruction revealed the occurrence of 35–36 full-sib families in the composite seed lot we tested. Also, consistent results were obtained by separate sampling in a 1st SNP assay using 108 SNPs and in a 2nd assay using 95 SNPs (Figure 3, Additional file 2: Table S7). The WWP breeding germplasm, comprising seed families selected from wild ecosystems, were confirmed to be strongly structured with complex populations. This current comprehensive genetic characterization contributes to the knowledge about levels and distribution of genetic diversity and gains novel insight into genetic subdivision within the available WWP resistance resources. Our results clarify knowledge of the genetic constitution of the collected *P. monticola* germplasm and could allow us to prioritize individuals on the basis of conservation value for minimizing loss of genetic variation in conservation program as well as to develop breeding recommendation with balance between maximizing gene diversity and minimizing inbreeding for tree improvement by identifying the main genitors. Genotypic data from our study may efficiently guide further application of this diversity in the long-term management and reforestation of this tree species across western North America.

## Conclusion

The present study represents the first research of candidate gene-based SNP discovery using pooled RNA-seq approach integrated with bulked segregation analysis in a five-needle pine. We generated novel transcriptome and SNP data from shoot-tip tissues of the *C. ribicola*-resistant and -susceptible WWP germplasm that originated from a composite seed lot. A subset of 432 SNP loci were verified by high-throughput genotyping and 52.5% of them were polymorphic. Using genotypic data of these SNP markers, parentage relationship and genetic diversity were determined in WWP germplasm collection and SNP markers were identified for breeding screening of resistance to WPBR across WWP populations. These validated SNP resources may open up new avenues for ecological genomics and comparative genetic mapping in five-needle pine species.

## Methods

### Plant material

A composite *P. monticola* seed lot with ‘major gene (*Cr2*) for HR-like resistance’ to WPBR was used in the present study. The lot was sourced mostly from parent trees that originated from the Champion Mine area on the Cottage Grove Ranger District of the Umpqua National Forest in Oregon. These parent trees comprised the breeding crosses were in the early fields established at the Dorena Genetic Resource Center (DGRC, Cottage Grove, Oregon); and the parents in those 1960’s grafts were heavily weighted toward Champion Mine parents (*Cr2/-*) and Bear Pass parents (many with *Cr2/-*) from the Bear Pass planting on the Willamette National Forest. There are also a few other clones in the breeding arboretum from other areas of Oregon and Washington.

Growing of seedlings, their artificial inoculation with *C. ribicola*, and phenotypic assessments were all performed at the DGRC, as described in Danchok et al. [51]. In brief, seeds were sown in June 2010 after four months stratification. Seedlings were grown in a green-house and inoculated with *C. ribicola* in September 2010 using infected leaves of Ribes spp. (the alternate host of *C. ribicola*) collected from locations outside of the geographical areas where the virulent isolate (*vcr2*) is known to occur. Inoculations were done using an average inoculum density of ~6,000 basidiospores/cm^2^ and a spore germination rate of 89%. Phenotypic traits were assessed at periodic intervals in 2011 when infection symptoms were evident on needles and stems. Each seedling was determined to be either a resistant (*Cr2/-)* or susceptible (*cr2/cr2*) genotype based both on their needle spot types (i.e., all HR-like; all susceptible; mixed; un-identified disease spots) and their stem symptoms (i.e., cankers present or absent). Needle samples were collected in July 2011 and stored at −20°C for genomic DNA extraction. In Oct. 2011 (~13 months post *C. ribicola*-infection), branch and stem tissues were collected from a sub-set of seedlings for each genotype using liquid nitrogen and stored at −80°C until RNA extraction.

### RNA-Seq analysis based on bulked segregation

Shoot tips from each of 45 resistant and 45 susceptible seedlings were collected individually and used for total RNA extraction following a protocol described previously [52]. RNA-seq analysis was performed by integration of bulked-segregation analysis. Total RNA samples were pooled into a total of six samples (each RNA sample was equally pooled from 15 seedlings): three with resistant (*Cr2/-)* phenotype and three with susceptible (*cr2/cr2*) phenotype. After DNase (RNase-free) treatment for 30 min at 37°C, mRNA was separated using an RNA-Seq sample preparation kit (Illumina) and used for construction of cDNA libraries as previously described [15] except each library contained sample-specific 6-bp nucleotide bar-coding tags. The six tagged cDNA libraries were pooled in equal ratios and used for 2 × 100 bp sequencing on one lane of the Illumina HiSeq2000 at the National Research Council of Canada (Saskaton, Canada). The raw Illumina RNA-seq 100-bp PE sequences were deposited in the NCBI SRA under accession number SRR1574690-1574692.

RNA-seq data analyses were performed using CLC Genomics Workbench 5.1 (CLC Bio, Cambridge, Mass, USA). Raw reads were trimmed before *de-novo* transcript assembly with default settings at quality limit = 0.05, ambiguous limit = 2, and minimum number of nucleotides in reads = 15. Shoot-tips of resistant (*Cr2/-)* seedlings were considered free of *C. ribicola* mycelia, so trimmed reads from the three cDNA libraries of resistant (*Cr2/-*) seedlings were *de-novo* assembled for generation of WWP shoot tip transcriptome with graph parameters of automatic word size and automatic bubble size and the parameters for mapping reads back to the contigs at mismatch cost = 2, length fraction = 0.5, similarity fraction = 0.8, deletion or insertion cost = 3, and minimum contig length = 200.

To verify *de novo* assembly quality, putative open reading frames (ORFs) within transcript sequences was identified by TransDecoder (http://transdecoder.sourceforge.net/) at minimum protein length of 50. Putative WWP protein sequences were compared with the PGI database (77,326 contigs, Release 9.0, March 26, 2011, http://compbio.dfci.harvard.edu/tgi/), and loblolly pine genome database (assembly v1.01, Nov. 20, 2013, http://pinegenome.org/). To estimate transcripts from infected *C. ribicola*, the WWP shoot-tip transcriptome was also search against the *M. laricis-populina* protein database (http://genome.jgi-psf.org/Mellp1/Mellp1.download.ftp.html).

### Contig annotation

As described in previous study [15], GO annotation assignment was performed against databases of the NCBI nr, PIR (http://pir.georgetown.edu/pirwww/), GO (http://www.geneontology.org/), UniProts (http://www.ebi.ac.uk/UniProt/), and KEGG (http://www.genome.jp/kegg/) using the BLAST2GO program (Biobam Bioinformatics S.L., Valencia, Spain, http://www.blast2go.com/). Annotation difference between WWP primary needle [15] and shoot-tip reference transcritptomes was assessed by the Fisher’s exact test with correction for multiple testing using BLAST2GO. Pinus HCGs in the WWP shoot-tip transcriptome were predicted using BLASTn against the PGI database with homology E values ≤ 10e-100. A set of WWP RGAs [10] and DEGs in host defense response to *C. ribicola* infection [15] were used to predict candidate genes expressed in shoot-tip tissues involved in genetic resistance against *C. ribicola* infection by BLAST search and sequence alignment analysis.

### SNP discovery and validation by high-throughput genotyping

*In-silico* SNP detection was performed by mapping RNA-seq PE reads of the six cDNA libraries back to the targeted sets of functional gene groups using CLC Genomics Workbench 5.1 with quality-based variation detection at the following parameters: window length = 11, maximum gap and mismatch count = 1, minimum average quality = 20, minimum central quality = 20, minimum coverage = 20, minimum variant frequency (MVF) = 30%, maximum expected variation (ploidy) = 2, and presence in both forward and reverse reads. Only reads that mapped to a single unique position on the reference sequences were used. To predict the effect of the mutation underlying each SNP at the amino acid level, the best ORFs predicted by TransDecoder were used for as reference sequences for SNP detection using CLC Genomics Workbench, and then each SNP was determined as a synonymous or non-synonymous mutation. SNPs in those WWP ORF sequences that showed best match to *P. taeda* and PGI databases at protein level by BLAST search were considered for SNP genotyping verification.

Due to unknown intron–exon boundaries and high proportion of paralogs in the pine gene families [53], additional, stringent criteria were considered when SNPs were selected for design of genotyping arrays. Criteria included contig SNP frequency, SNP locations and flanking sequence on the 3′- and 5′- ends. For SNP discovery in a candidate gene approach, *in-silico* SNP data were generated using HCGs, DEGs, and RGAs as separate mapping references.

For SNP genotyping, genomic DNA was extracted from needle tissues of individual seedlings belonging to the same composite seed lot used in RNA-seq analysis. About 100 mg of needle tissues were cut into small fragments and homogenized in liquid nitrogen using a FastPrep®-24 Instrument (MP Biomedicals, Santa Ana, CA, USA). Genomic DNA was extracted using a DNeasy Plant Mini kit (Qiagen, Mississauga, ON, Canada).

High-throughput genotyping was conducted using the Sequenom iPlex MassARRAY platform (Sequenom, San Diego, CA, USA) [54] at the Génome Québec Innovation Centre, McGill University. Two SNP assays were designed separately, each composed of 216 SNP loci and genotyped in a collection of 188 seedlings (~50% resistant and ~50% susceptible samples). Almost every SNP was selected from a unique functional gene except that the 2nd SNP array contained 20 SNPs from six genes with two to four SNPs in the same contig. Multiplex assays were designed using the MASSARRAY® Assay Design software for 36 SNPs in each of six multiplex panels set with the following parameters: amplicon length (bp): min:80, optimum:120, max:320; PCR primer length (bp): min:16, optimum:20, max:25; extension primer length (bp): min: 16, max: 31; hybridization Tm (°C): min: 45, max:100. PCR reactions were performed using Sequenom iPlex Gold reagent kits following standard procedures. About 20 ng of genomic DNA was amplified using a pool of 36 pairs of PCR primers under cycling conditions at 95°C for 15 min, 45 × (95°C for 20 sec, 56°C for 30 sec, 72°C for 60 sec), and final extension at 72°C for 3 min. The shrimp alcaline phosphatase was used to remove all unincorporated dNTPs. After single base extension for probes, the products were spotted on a Sequenom 384-well chip using a Nanodispenser and the chip was read by a Mass Spectrometer. Genotypes for each SNP marker in each sample were analyzed by the MassARRAY Analyzer 4 System. Sequence and nucleotide variation of verified SNP markers have been submitted to GenBank dbSNP databases (GenBank: ss#947846159 – ss#947846384).

### SNP genotypic data analysis

The quality of SNP genotyping was manually assessed for each SNP locus in the sample collection. Population characteristics of the SNPs such as MAF, Ho, He, and the deviation from HWE were calculated using GenAlex 6.41 [55]. SNPs with a call rate below 80% of the total samples, a MAF below 0.05, and a rate of heterozygosity below 5% were excluded for further analysis.

PCA and Bayesian phylogenetic methods were used to identify if there was any population structure/grouping in the composite seed lot. SNP data were converted into allele frequencies based on SNP genotype of each individual seedlings and PCA was performed using the variance-covariance matrix of SNP allele frequencies in TASSEL [56]. Seedlings were assigned to ancestry clusters using the Bayesian model-based clustering algorithm by assuming Hardy-Weinberg equilibrium and linkage equilibrium within populations in the software package STRUCTURE [57]. The no-admixture model, which assumes that each individual comes from only one of the clusters, was used for the SNP haplotype analysis with 50,000 burn-in length and 500,000 replicates. Twenty simulation runs were performed with K values set from 1 to 10 to estimate the cluster number (K). The most likely number of clusters was then determined using the DeltaK method [58].

Individual assignment by STRUCTURE analysis may group different seed families into one population. Sibship analysis and parentage reconstruction of the WWP gemplasm were conducted using the most accurate full-pedigree likelihood method of the COLONY program [59].

Chi-squared test was used to evaluate LD between all pair-wise combinations of SNPs. Association of SNP loci with phenotypic traits of major gene (*Cr2*) resistance in WWP was evaluated by looking at LD values (*r*^*2*^) among SNP sites and resistance phenotypes (*Cr2/-* vs. *cr2/cr2*) using TASSEL as described previously [11]. For multiple SNPs, a weighted average value of *r*^*2*^ was calculated between each SNP pair [60]. *P*-values for *r*^*2*^ were adjusted using a Bonferroni correction [61].

### Availability of supporting data

The data sets supporting the results of this article are included within the article and its additional supporting information files. Illumina raw sequences were deposited in the NCBI GenBank SRA under accession number SRR1574690-1574692. The WWP shoot-tip reference transcriptome with 54,661 contigs was deposited at GenBank under accession GBQX01000000. SNP markers were deposited in GenBank under accession numbers ss#947846159 – ss#947846384.

## Additional files

Additional file 1: Table S1. Characteristics of the shoot-tip transcriptome *de novo* assembled from RNA-seq reads of three cDNA libraries constructed from resistant seedlings. **Table S2.** Putative *Pinus monticola* proteins (with minimum length of 50) predicted by TansDecoder. **Table S3.** BLAST analysis of the western white pine shoot-tip reference transcriptome. **Table S4.** Summary of SNPs detected in three groups of candidate genes in the shoot-tip reference transcriptome by mapping of RNA-seq reads with highly stringent thresholds. Additional file 2: Figure S1. BLAST top-hit species distribution. Additional file 3: Figure S2. Differential GO-term distribution between reference transcriptomes of western white pine shoot-tip and primary needle. Fisher’s exact test was performed using program BLAST2GO with term filter value at 0.05 and term filter mode of corrected p-value by false discovery rate (FDR) control. Categories of GO terms include cell components C1, plasma membrane; C2, cytosol; C3, extracellular region; C4, cell wall; C5, ribosome; C6, endoplasmic reticulum, C7, thylakoid; C8, nucleolus; C9, endosome; C10, cytoskeleton; C11, plasmodesma; C12, nucleoplasm; C13, vacuolar membrane; C14, trans-Golgi network; C15, viral nucleocapsid; F1, RNA binding; F2, chromatin binding; F3, oxidoreductase activity, acting on paired donors, with incorporation or reduction of molecular oxygen; F4, carbohydrate binding; F5, receptor binding; F6, ADP binding; F7, hydroquinone:oxygen oxidoreductase activity; F8, protein kinase binding; F9, RNA polymerase II transcription cofactor activity; F10, catechol O-methyltransferase activity; F11, aminoacyl-tRNA editing activity; P1, lipid metabolic process; P2, response to endogenous stimulus; P3, anatomical structure morphogenesis; P4, response to biotic stimulus; P5, reproduction, P6, DNA metabolic process; P7, translation; P8, cell differentiation; P9, cell cycle; P10, flower development; P11, generation of precursor metabolites and energy; P12, embryo development; P13, cell growth; P14, secondary metabolic process; P15, regulation of gene expression, epigenetic; P16, photosynthesis; P17, response to extracellular stimulus; P18, pollination; P19, tropism; P20, methylation; P21, ATP catabolic process; P22, cell-cell signaling; P23, response to karrikin; P24, pectin catabolic process; P25, regulation of plant-type hypersensitive response; P26, membrane fusion; P27, MAPK cascade; P28, Golgi organization; P29, cellular response to iron ion; P30, protein peptidyl-prolyl isomerization; P31, carbohydrate transmembrane transport; P32, amino acid transmembrane transport; P33, response to cyclopentenone; P34, monocarboxylic acid transport. Additional file 4: Table S5. Sequences of primers and probes used for Sequenom iPlex genotyping on the SNPs with successfully verified genotypes in western white pine populations. **Table S6.** Population genetic parameters of the SNPs with successfully verified genotypes. **Table S7.** Parentage analysis for assignment of the best full-sib families in the resistance germplasm using Colony software package. Additional file 5: Figure S3A. Distributions of minor allele frequency (MAF) and observed heterozygosities (Ho) of the SNP loci genotyped successfully with polymorphism and call rate > 80% in the whole array set. (A) Distributions of minor allele frequency (MAF). Additional file 6: Figure S3B. Distributions of minor allele frequency (MAF) and observed heterozygosities (Ho) of the SNP loci genotyped successfully with polymorphism and call rate > 80% in the whole array set. (B) Distributions of observed heterozygosities (Ho). Additional file 7: Figure S4. Graph of the first three principal components based on marker frequencies. Principle component analysis (PCA) was based on SNP genotypic data showing genetic diversity of a composite seed lot from a western white pine breeding program. Additional file 8: Figure S5. Estimated number of clusters obtained with STRUCTURE for K values from 1 to 10 using SNP data. Graphical representations are shown the statistics ΔK in two SNP arrays separately by STRUCTURE simulations. (A) The ΔK was calculated based on genotypic data of 108 SNP markers in 179 samples of the 1st SNP array; and (B) The ΔK was calculated based on genotypic data of 95 SNP markers in 188 samples of the 2nd SNP array.

## Abbreviations

AFLP: Amplified fragment length polymorphism
DEG: Differential expressed genes in plant defense response
He: Expected levels of heterozygosity
HEW: Hardy–Weinberg equilibrium
Ho: Observed levels of heterozygosity
LD: Linkage disequilibrium
MAF: Minor allele frequency
NBS-LRR: Nucleotide-binding site and leucine-rich repeat
PCA: Principal component analysis
HCG: Pinus highly conserved gene
RGA: Resistance gene analog
RLK: Receptor-like protein kinase
SNP: Single nucleotide polymorphism
SSR: Simple sequence repeat
WPBR: White pine blaster rust
WWP: Western white pine
