# Supplementary figures and images for: Western white pine SNP discovery and high-throughput genotyping for breeding and conservation applications
Source: BMC Plant Biol. 2014 Dec 30;14:380. doi: 10.1186/s12870-014-0380-6 (PMC4302426; doi:10.1186/s12870-014-0380-6)

## Slide 1
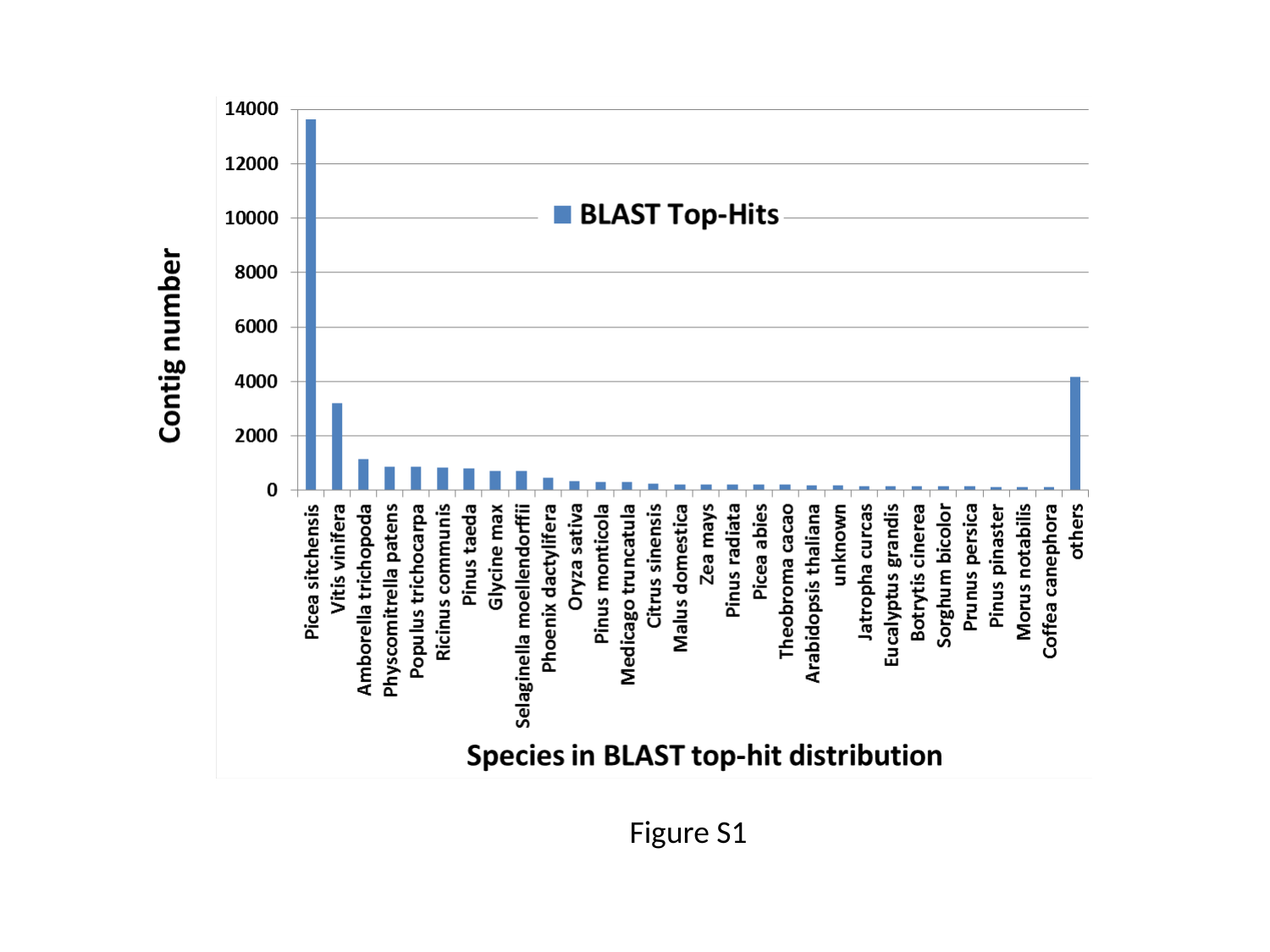

Figure S1

Supplement: Additional file 3: Figure S2. — Differential GO-term distribution between reference transcriptomes of western white pine shoot-tip and primary needle. Fisher’s exact test was performed using program BLAST2GO with term filter value at 0.05 and term filter mode of corrected p-value by false discovery rate (FDR) control. Categories of GO terms include cell components C1, plasma membrane; C2, cytosol; C3, extracellular region; C4, cell wall; C5, ribosome; C6, endoplasmic reticulum, C7, thylakoid; C8, nucleolus; C9, endosome; C10, cytoskeleton; C11, plasmodesma; C12, nucleoplasm; C13, vacuolar membrane; C14, trans-Golgi network; C15, viral nucleocapsid; F1, RNA binding; F2, chromatin binding; F3, oxidoreductase activity, acting on paired donors, with incorporation or reduction of molecular oxygen; F4, carbohydrate binding; F5, receptor binding; F6, ADP binding; F7, hydroquinone:oxygen oxidoreductase activity; F8, protein kinase binding; F9, RNA polymerase II transcription cofactor activity; F10, catechol O-methyltransferase activity; F11, aminoacyl-tRNA editing activity; P1, lipid metabolic process; P2, response to endogenous stimulus; P3, anatomical structure morphogenesis; P4, response to biotic stimulus; P5, reproduction, P6, DNA metabolic process; P7, translation; P8, cell differentiation; P9, cell cycle; P10, flower development; P11, generation of precursor metabolites and energy; P12, embryo development; P13, cell growth; P14, secondary metabolic process; P15, regulation of gene expression, epigenetic; P16, photosynthesis; P17, response to extracellular stimulus; P18, pollination; P19, tropism; P20, methylation; P21, ATP catabolic process; P22, cell-cell signaling; P23, response to karrikin; P24, pectin catabolic process; P25, regulation of plant-type hypersensitive response; P26, membrane fusion; P27, MAPK cascade; P28, Golgi organization; P29, cellular response to iron ion; P30, protein peptidyl-prolyl isomerization; P31, carbohydrate transmembrane transport; P32, amino acid transmembrane trans [file 12870_2014_380_MOESM3_ESM.pptx]

## Slide 1
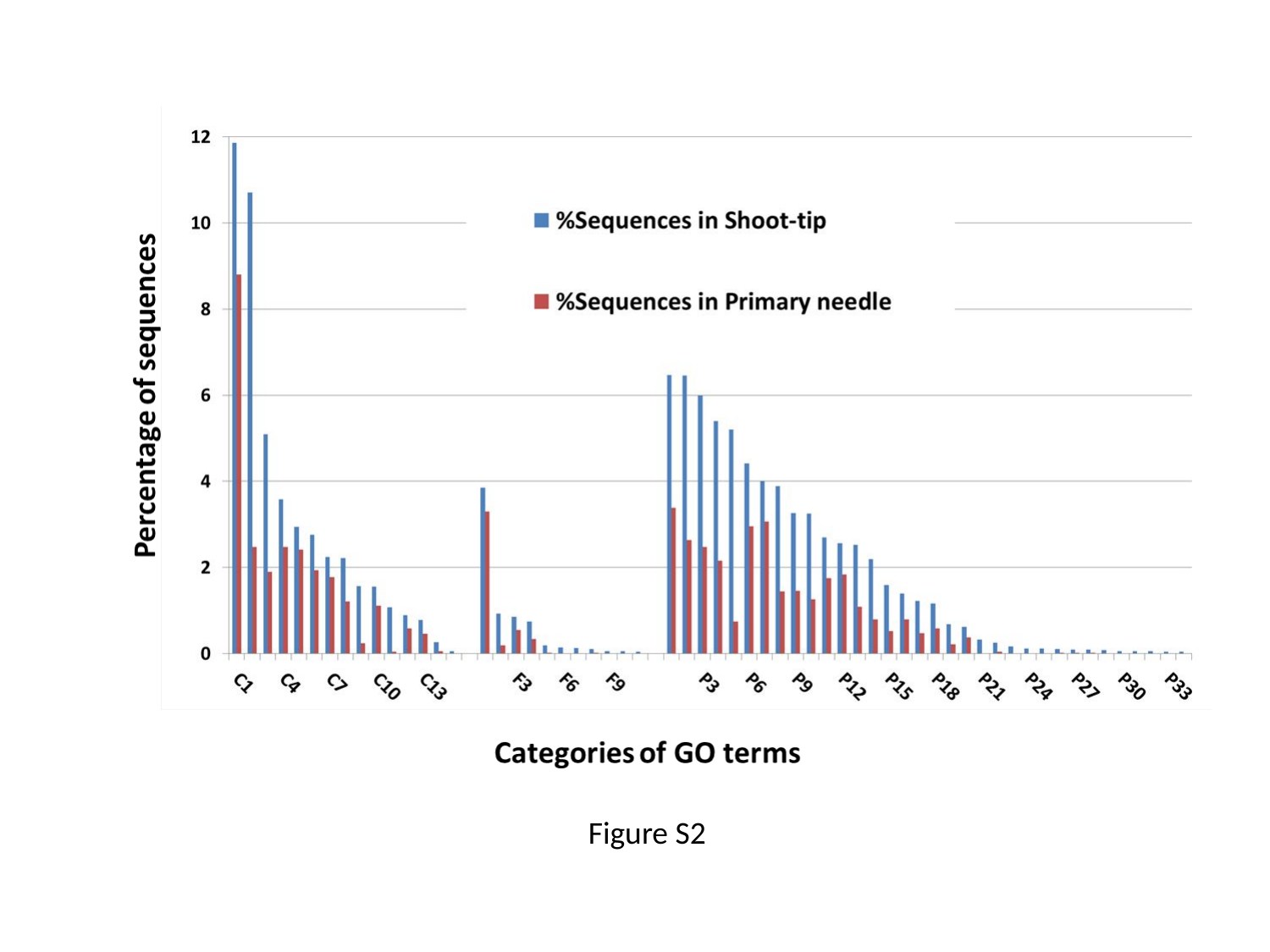

Figure S2

Supplement: Additional file 4: Table S5. — Sequences of primers and probes used for Sequenom iPlex genotyping on the SNPs with successfully verified genotypes in western white pine populations. Table S6. Population genetic parameters of the SNPs with successfully verified genotypes. Table S7. Parentage analysis for assignment of the best full-sib families in the resistance germplasm using Colony software package. [file 12870_2014_380_MOESM4_ESM.pptx]

## Slide 1
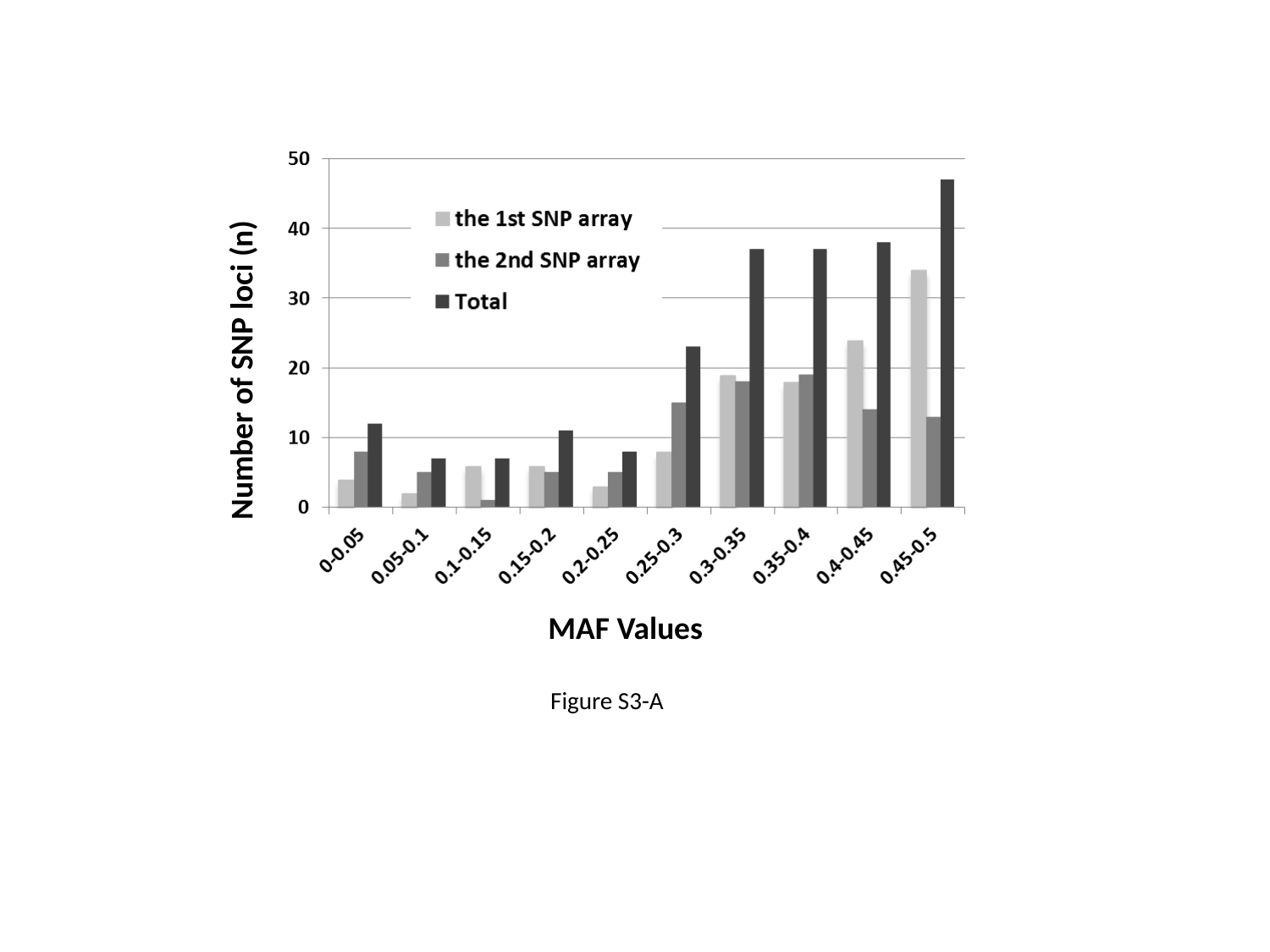

Number of SNP loci (n)
MAF Values
Figure S3-A

Supplement: Additional file 5: Figure S3A. — Distributions of minor allele frequency (MAF) and observed heterozygosities (Ho) of the SNP loci genotyped successfully with polymorphism and call rate > 80% in the whole array set. (A) Distributions of minor allele frequency (MAF). [file 12870_2014_380_MOESM5_ESM.pptx]

## Slide 1
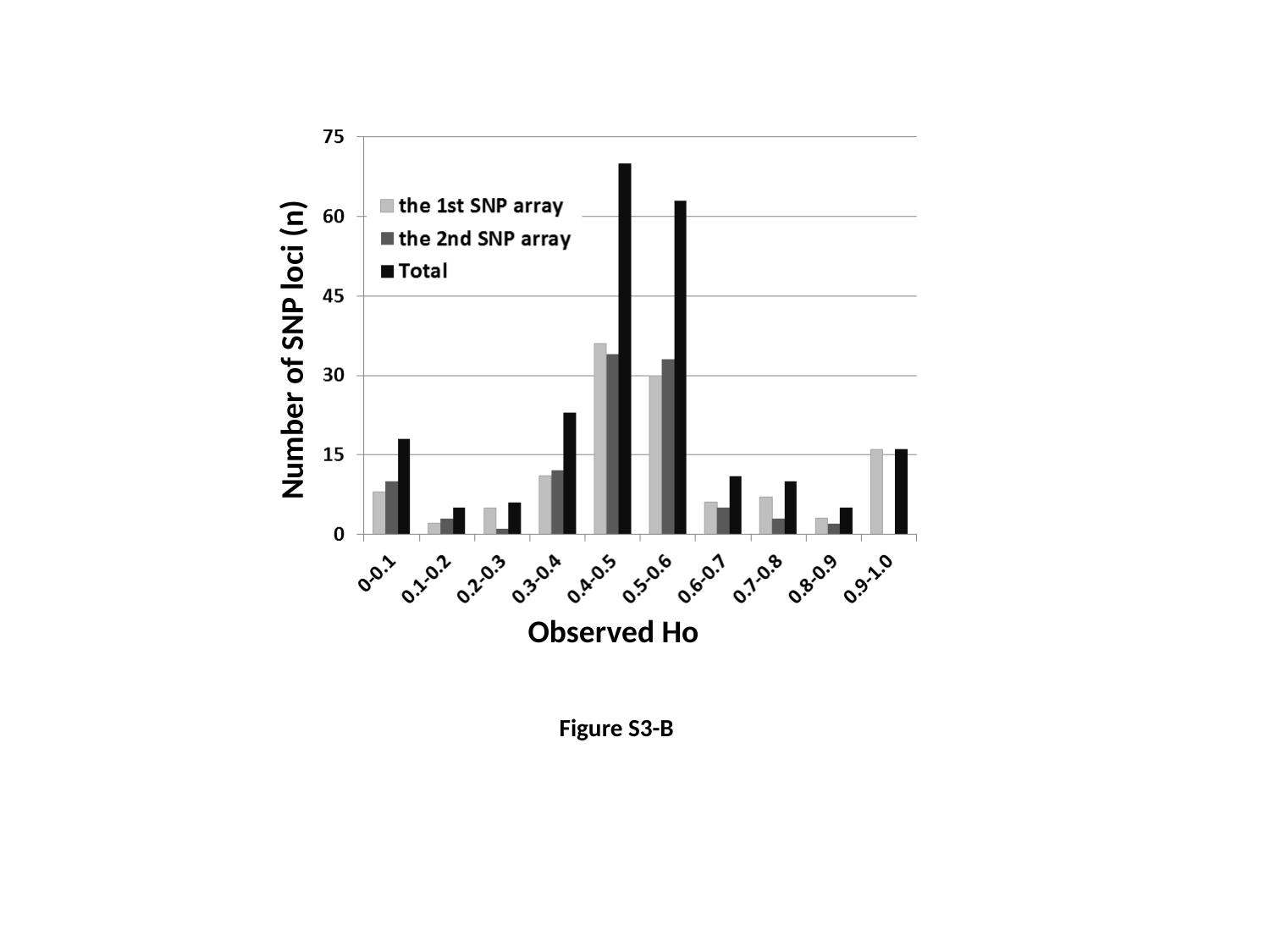

Number of SNP loci (n)
Observed Ho
Figure S3-B

Supplement: Additional file 6: Figure S3B. — Distributions of minor allele frequency (MAF) and observed heterozygosities (Ho) of the SNP loci genotyped successfully with polymorphism and call rate > 80% in the whole array set. (B) Distributions of observed heterozygosities (Ho). [file 12870_2014_380_MOESM6_ESM.pptx]

## Slide 1
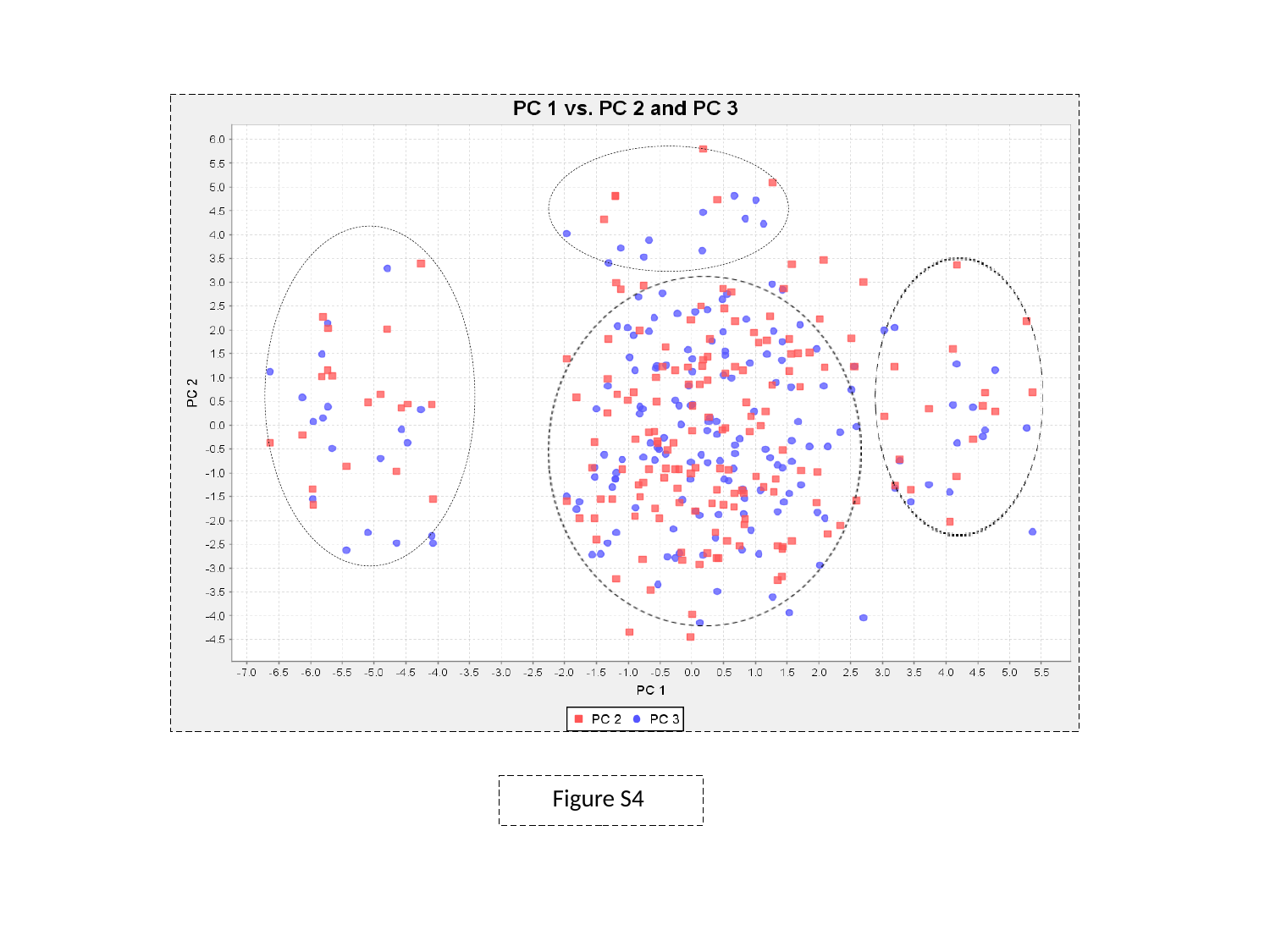

Figure S4

Supplement: Additional file 7: Figure S4. — Graph of the first three principal components based on marker frequencies. Principle component analysis (PCA) was based on SNP genotypic data showing genetic diversity of a composite seed lot from a western white pine breeding program. [file 12870_2014_380_MOESM7_ESM.pptx]
