# Supplementary material for: Western white pine SNP discovery and high-throughput genotyping for breeding and conservation applications
Source: BMC Plant Biol. 2014 Dec 30;14:380. doi: 10.1186/s12870-014-0380-6 (PMC4302426; doi:10.1186/s12870-014-0380-6)
Supplement: Additional file 8: Figure S5. — Estimated number of clusters obtained with STRUCTURE for K values from 1 to 10 using SNP data. Graphical representations are shown the statistics ΔK in two SNP arrays separately by STRUCTURE simulations. (A) The ΔK was calculated based on genotypic data of 108 SNP markers in 179 samples of the 1st SNP array; and (B) The ΔK was calculated based on genotypic data of 95 SNP markers in 188 samples of the 2nd SNP array. [file 12870_2014_380_MOESM8_ESM.pptx]

## Slide 1
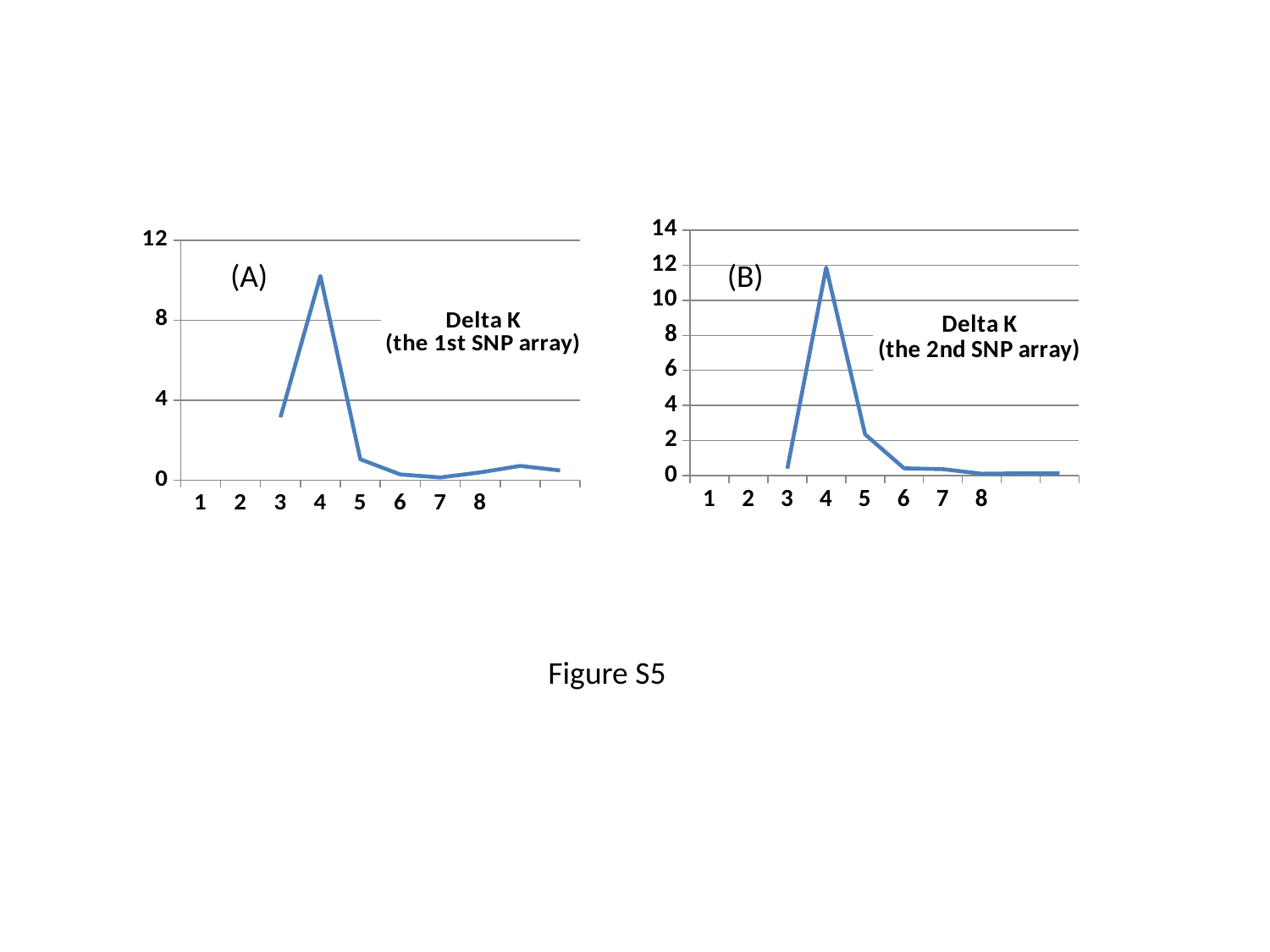

### Chart: Delta K
(the 2nd SNP array)
| Category | Delta K |
|---|---|
### Chart: Delta K
(the 1st SNP array)
| Category | Delta (K) |
|---|---|(A) (B)
Figure S5
